# Accelerated Aging in HIV Patients

**DOI:** 10.5041/RMMJ.10089

**Published:** 2012-10-31

**Authors:** Keren Meir-Shafrir, Shimon Pollack

**Affiliations:** 1 Institute for Allergy, Immunology & AIDS, Rambam Health Care Campus, Haifa, Israel, and the; 2 Ruth and Bruce Rappaport Faculty of Medicine, Technion-Israel Institute of Technology, Haifa, Israel

**Keywords:** Aging, AIDS, HIV, immune senescence

## Abstract

Life expectancy has been increasing in the last few decades in the Western world and is accompanied by higher occurrence of age-related diseases like metabolic, cardiovascular, and renal diseases and also with a decline in immune functions. In HIV-infected people, due to the use of combination antiretroviral therapy (cART), life expectancy has increased. As a result, non-AIDS conditions which are age-associated have become more prevalent and appear earlier, resulting in accelerated aging in HIV patients. These non-AIDS conditions in HIV patients are associated with CD4+ T cell counts: lower counts are associated with higher rates of liver, cardiovascular, renal, and neurocognitive disorders. The effect of viral load and cART on the earlier occurrence of age-associated diseases is less significant than the CD4 count effect. Thus, the loss of immune functions in HIV-infected patients may enhance aging.

HIV/AIDS came to the world’s awareness over 30 years ago, with the first reports of young homosexual men, considered to be previously healthy, suffering from various types of opportunistic infections and profound cellular immunodeficiency.^1^ In the relatively short time since then, it has grown in scale to become a worldwide epidemic, with an estimated number of 34 million people living with HIV by 2011.^2^ The introduction of antiretroviral therapy (ART) in the middle of the 1990s has improved survival with HIV dramatically (Figure 1) and turned HIV infection, in effect, into a chronic condition. The percentage of people living with HIV/AIDS in USA who are over 50 years old is on the rise (Figure 2), and it is estimated that, by 2015, people over 50 will constitute the majority of all people living with HIV/AIDS in USA.^5^

**Figure 1:**
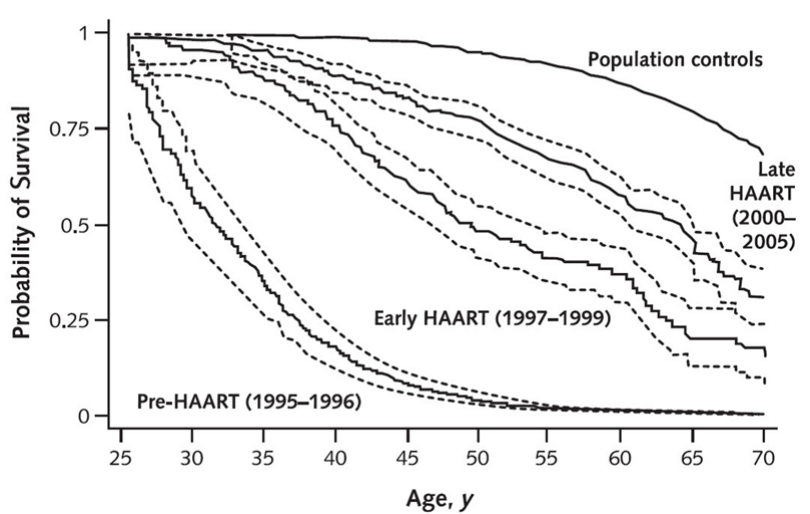
**Estimated survival of 25-year-old HIV-infected and non-infected men in Denmark, 1995–2005.** Persons with HIV infection are divided into 3 calendar periods of observation. Dashed lines indicate 95% CIs. HIV = human immunodeficiency virus; HAART = highly active antiretroviral therapy. Reprinted from Lohse et al.^3^ with permission of the American College of Physicians.

**Figure 2:**
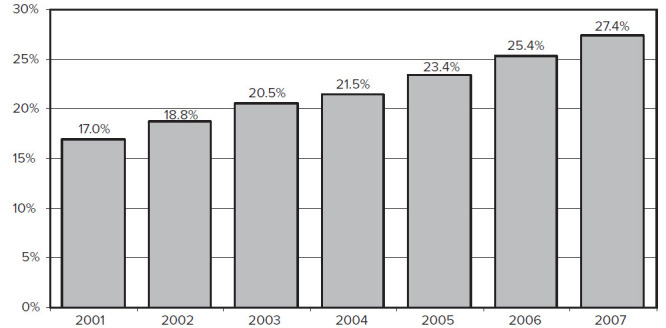
**Estimated percentage of persons living with HIV/AIDS in USA who are older than 50, by year, 2001–2007.** Modified from *Growing Older with the Epidemic: HIV and Aging*,^4^ with permission.

As a result, we are encountering more chronic diseases typical of aging: cardiovascular disease, diabetes mellitus, dyslipidemia, osteoporosis and bone fractures, malignancies, and neurocognitive impairment.^6^ In addition, the accelerated aging of the immune system of HIV carriers has been demonstrated,^7^ and this is accompanied by the parallel process of increased incidence of chronic diseases typical of aging and early signs of physical and functional frailty in this population.^8^

Accelerated aging may be a result of several factors, including HIV infection itself, ART side-effects, and the aging of the immune system. It is now clear that the function of the immune system declines with age, but is the decline affecting the accelerated aging in HIV patients?

These evolving processes which interact with each other are becoming a major factor in treatment decisions of HIV carriers and shape research and clinical priorities, and they will be discussed further in this review.

## IMMUNOSENESCENCE AND HIV INFECTION

Physiological aging of the immune system, termed immune senescence, is associated with a dysfunction in innate and adaptive immunity which diminishes the ability to respond to novel foreign antigens—vaccinations and infections. Similar changes in immune functions occur in people with chronic HIV infection but at a much younger age.

Changes seen in adaptive immune system manifest as lower naïve:memory CD4 ratio and enrichment of CD28−/CD57+/CD8+ effector T cells.^9^ The latter are senescent cells with shorter telomeres and limited proliferative capacity. In addition, there are putative qualitative and quantitative changes in T regulatory cells^10^ and a decrease in the diversity of naïve B cells and a qualitative B cell dysfunction.^11^

In HIV carriers, peripheral blood lymphocytes show a tendency towards T cell senescence with enrichment of CD28−/CD57+/CD8+ T cells and inverted ratio of naïve/memory T cells,^9^ as seen in normal aging. However, the immunophenotypic changes seen in HIV-infected patients, though similar to the changes seen in HIV-negative individuals, appear 20–30 years earlier.^12^ Considering the innate immune system, peripheral blood monocytes from young HIV-positive individuals exhibit changes in phenotype, function, and telomere length that closely resemble those observed in elderly controls aged approximately 30 years older. Furthermore, these immune defects are not fully restored by ART.^13^ Another example of the same accelerated aging process is the telomere length of CD4+ and CD8+ T cells in HIV carriers, which resembles the telomere length of HIV-negative patients 38 years older.^14^

Chronic activation of the immune system probably contributes to the accelerated aging in HIV-infected patients.^15^ The chronic inflammatory process caused by the persistent immune activation is associated with the increased release of pro-inflammatory cytokines such as IL-6, IL-1β, and TNF-α as well as pro-coagulants such as cystatin-C and D-dimer.^16^ These plasma biomarkers of inflammation decline dramatically with combination antiretroviral therapy (cART) administration, but do not normalize entirely.^17^ Chronic inflammatory manifestations are also seen in physiological aging and have been implicated in the development of cardiovascular disease in aged people. Chronic inflammation may also serve as a proximate mediator to functional decline,^18^ and to frailty development in aging.^19^

## NON-AIDS COMPLICATIONS IN AGING HIV-INFECTED PATIENTS

One of the important studies of the ART era was the Strategies for Management of Antiretroviral Therapy (SMART).^20^ It was designed to compare two treatment strategies: one which was viral suppressive and continuous regardless of CD4 count; the other with treatment interruptions according to CD4 levels. After a mean follow-up period of 16 months, the study review board recommended to stop enrollment to the trial because of a safety risk in the treatment interruption group. The statistical analysis showed that patients in the interruption group had an increased risk of mortality, both from opportunistic infections and from cardiovascular, renal, or hepatic disease. This study demonstrated the health effects of HIV beyond AIDS-defining illnesses.

## METABOLIC CHANGES AND CARDIOVASCULAR DISEASE

The HIV-positive population experiences both external and internal metabolic changes. Abnormal fat distribution, also known as lipodystrophy, occurs in both treated^21^ and untreated^22^ HIV-positive patients. It includes two different syndromes: lipoatrophy, or subcutaneous fat loss of face, extremities, and buttocks; and lipohypertrophy, or central fat deposition, manifested as intra-abdominal (visceral) fat, buffalo hump, or breast enlargement. The risk factors for the two abnormal fat distribution syndromes are different. According to the Fat Redistribution and Metabolic Change in HIV Infection (FRAM) study, lipoatrophy can be found in almost 40% of HIV-positive men^23^ and 30% of HIV-positive women.^24^ Patients at higher risk to develop lipoatrophy are the ones with lower BMI (body mass index), higher nadir HIV load, and use of ART, especially stavudine, zidovudine, and earlier protease inhibitors (PIs).^25^ Lipohypertrophy is more common in HIV-positive women than HIV-positive men and in individuals with greater body fat levels to begin with.^26^

HIV-positive patients with abnormal fat distribution have significantly increased prevalence of dyslipidemia and impaired glucose homeostasis in comparison with HIV-negative controls matched for age and BMI.^27^ The dyslipidemia associated with HIV infection itself includes elevated triglyceride levels and decreased high-density lipoprotein cholesterol (HDL-C) levels. ART is also a major contributor to dyslipidemia, mainly a more profound elevation of triglycerides with ritonavir-based PI regimens.^28^ Likewise, both decreased subcutaneous leg fat and increased visceral fat are strongly associated with decreased insulin sensitivity in this population.^29^ In addition, ART may have an effect on insulin sensitivity, mainly the PIs. One of the mechanisms by which PIs induce insulin resistance is through blocking the transport of glucose by the insulin-sensitive glucose transporter GLUT4.^30^ A prospective 10-year follow-up of 1,046 ART-treated HIV-positive patients demonstrated an increased incidence of diabetes mellitus in comparison to the general population, and the risk factors were older age, adiposity, and short exposure to the PI indinavir and the nucleoside reverse transcriptase inhibitors (NRTIs) stavudine and didanosine,^31^ which are mostly not used today in the developed world.

The combination of metabolic and immunologic changes are the base of cardiovascular disease (CVD) in HIV-positive patients.^32^ In addition to the established risk factors for coronary heart disease (CHD) in the general population, which have been shown to be increased in the HIV-positive population,^33^ there is additional risk that might be explained in part by both antiretroviral medications and novel CHD risk factors including inflammation and immune dysfunction. The effect of ART was assessed in the Data Collection on Adverse Events of Anti-HIV Drugs (DAD) study, which demonstrated an association between duration of exposure to combination ART and the risk of myocardial infarction, specifically with exposure to PIs.^34^ In contrast, a large study from the Veteran Affairs (VA) system showed no connection between any ART class and CHD or cerebrovascular event outcomes.

Several surrogate indices of CVD have been tested in HIV-positive patients. A recent study demonstrated an association between immune activation markers and carotid artery plaque in patients virologically suppressed on ART, and another study demonstrated elevated carotid intima-media thickness in all HIV groups versus controls, including elite-controllers (HIV-infected patients who maintain an undetectable HIV RNA by standard assay in the absence of ART).^35^ The same trend was demonstrated with increased prevalence of subclinical coronary atherosclerosis detected by coronary computed tomography angiography in HIV-infected men in comparison with controls.^36^

The actual increased risk for CHD and acute myocardial infarction in HIV-positive patients was shown in several studies, which found significantly increased risk ratios up to 1.94 (95% CI 1.58–2.37).^37^

### Renal Complications

The pathogenesis of renal disease in HIV-positive individuals is diverse. It includes: 1) HIV-associated nephropathy (HIVAN), a form of focal segmental glomerulosclerosis that is accompanied by tubuleinterstitial inflammation, and clinically manifests as rapidly progressive renal failure with nephritic range proteinuria. 2) HIV immune complex kidney disease (HIVICK), a collective term that includes IgA nephropathy, membranoproliferative glomerulonephritis, membranous nephropathy, and a lupus-like glomerulonephritis that is serologically negative.^38^ 3) Hypertensive and atherosclerotic renal disease. 4) ART side-effects, mainly tenofovir-induced renal tubular injury^39^ and indinavir/atazanavir-induced crystaluria and renal calculi formation.^40^ The first two pathologies are more common in untreated patients, the last two in treated. It has been shown that chronic kidney disease and proteinuria are associated with increased risk of mortality in HIV-positive patients.^41^

### Bone Mineral Density and Osteoporosis

Several population-based studies in the United States showed increased prevalence of osteoporotic fractures in HIV-infected men and women compared with HIV-uninfected individuals.^42^ The etiology of low bone mineral density (BMD) in HIV-positive patients is multifactorial. It includes both traditional, non-HIV-related risk factors such as smoking, alcohol and opiate use, low body weight, and vitamin D deficiency; and also HIV-related factors such as direct viral and inflammatory effects on bone resorption^43^,^44^ and the effects of ART, especially tenofovir.^45^ Multiple studies have shown a 2%–6% BMD loss after 48–96 weeks of therapy, regardless of the type of ART initiated.^46^ Several longitudinal studies have shown that, with continued ART use, BMD stabilizes over time.^47^,^48^

### Neurocognitive Changes

HIV-associated neurocognitive disorder (HAND) is divided into three levels of impairment: asymptomatic neurocognitive impairment, mild neurocognitive disorders, and HIV-associated dementia (HAD). The introduction of ART has reduced significantly the rate of HAD, but unfortunately the effect on less severe forms of impairment is not as impressive. Studies of HAND in treated patients have documented high persisting rates of mild-to-moderate neurocognitive impairment despite effective suppressing antiretroviral treatment,^49^ especially in individuals with a history of low nadir CD4s.^50^

### Frailty Syndrome in HIV-positive Older Adults

Frailty is defined as a syndrome of decreased physiological reserve, which increases vulnerability to negative outcomes such as loss of independence, nursing home admission, morbidity, and mortality.^51^ Recent studies demonstrated that HIV-positive individuals are at an increased risk of frailty and that some individuals with HIV manifest frailty characteristics at a much younger age than frail individuals without HIV.^6^ In the pre-ART era frailty in HIV was connected to the AIDS-wasting syndrome, with advanced immunosuppression and very high viral loads. In contrast, the current risk factors for frailty in the HIV-positive population is high fat mass, particularly trunkal fat, and high BMI.^52^

## CONCLUSION

Accelerated aging of the immune system together with earlier appearance of aging co-morbidities (Figure) in HIV patients point to a potential major contribution of immune system dysfunction to the accelerated aging in HIV-infected patients. This may once again highlight the role of normal immune function as a critical factor in the fight against HIV which, if successful, may both suppress HIV and also attenuate the process of accelerated aging. Successful cART is critical to the recovery of the immune system in HIV-infected individuals. Early initiation of antiretroviral therapy once HIV diagnosis has been established, which will probably keep the normal function of the immune system, may help in alleviating at least some of the morbid conditions related to accelerated aging. We will be able to verify this hypothesis once the results of the on-going international large study, testing the right time to start cART (START study), come out.^54^

**Figure 3:**
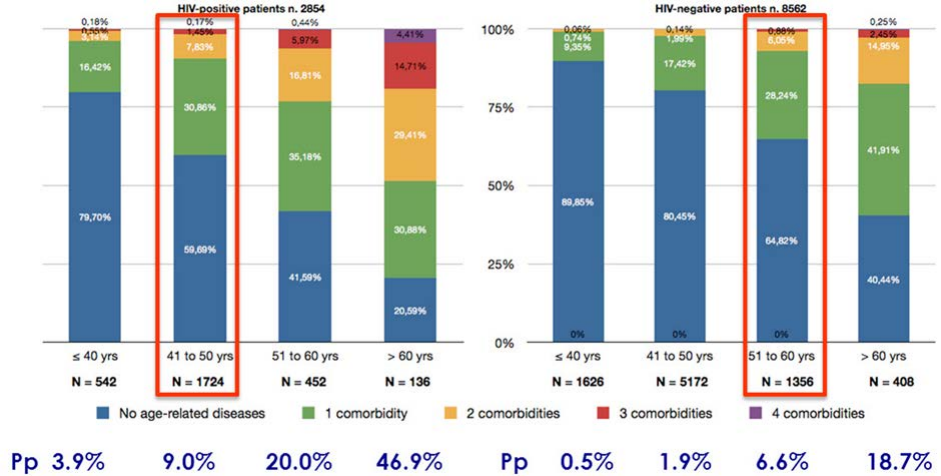
**Poly-patology (Pp) prevalence of age-related non-AIDS conditions in HIV-positive versus HIV-negative populations, 2002–2008.** Modified from Guaraldi G et al.,^53^ with permission.

## Abbreviations:

ART: antiretroviral therapy;
BMD: bone mineral density;
BMI: body mass index;
cART: combination antiretroviral treatment;
FRAM: Fat Redistribution and Metabolic Change in HIV Infection;
HAD: HIV-associated dementia;
HAND: HIV-associated neurocognitive disorder;
HIVAN: HIV-associated nephropathy;
HIVICK: HIV immune complex kidney disease;
NRTIs: nucleoside reverse transcriptase inhibitors;
SMART: Strategies for Management of Antiretroviral Therapy.
